# The Importance of Biochemical Screenings in the Diagnosis of Hypophosphatasia: Applications, Methodologies, and Challenges

**DOI:** 10.3390/ijms27031144

**Published:** 2026-01-23

**Authors:** Francesca Marini, Gaia Palmini, Simone Donati, Francesca Giusti, Maria Luisa Brandi

**Affiliations:** 1 FIRMOLab 2.0—Italian Foundation for Research on Bone Diseases (Fondazione FIRMO Onlus), 50129 Florence, Italy; gaia@fondazionefirmo.com (G.P.); marialuisa@marialuisabrandi.it (M.L.B.); 2 Department of Experimental and Clinical Biomedical Sciences, University of Florence, 50139 Florence, Italy; simone.donati@unifi.it; 3 Donatello Bone Clinic, Villa Donatello Hospital, 50019 Sesto Fiorentino, Italy; francesca.giusti@unifi.it; 4 Stabilimento Chimico Farmaceutico Militare (SCFM), 50141 Florence, Italy

**Keywords:** hypophosphatasia (HPP), tissue-non-specific alkaline phosphatase (TNSALP), total alkaline phosphatase (ALP), pyridoxal 5′-phosphate (PLP), active form of vitamin B6, phosphoethanolamine (PEA), inorganic pyrophosphate (PPi)

## Abstract

Pathological reduction in enzymatic activity of the tissue-non-specific alkaline phosphatase (TNSALP) is the molecular hallmark of hypophosphatasia (HPP), a group of rare inborn systemic diseases, mainly characterized by pathological affections of calcified tissue mineralization and the musculoskeletal system. The disease, in all clinical forms, is biochemically characterized by variable degrees of chronically reduced activity of circulating total alkaline phosphatase (ALP). Repeated detection of low values of ALP activity is mandatory to diagnose the presence of HPP, but, alone, it is not sufficient for the diagnosis of the disease. Detection of increased circulating levels of one of the main natural substrates of TNSALP, the pyridoxal 5′-phosphate (PLP), is needed to biochemically confirm the diagnosis of HPP. Urinary and/or blood levels of phosphoethanolamine (PEA) and inorganic pyrophosphate (PPi), two other natural substrates of TNSALP, can be elevated in a percentage of HPP patients. The contemporary biochemical evaluation of ALP activity and its target substrates is of great help in the diagnosis of HPP, and also for the monitoring of a patient’s response to enzymatic replacement therapy or other pharmacological treatments. Here, we describe and discuss possibilities and challenges of biochemical screenings for HPP, based also on the experience gained in our analysis laboratory.

## 1. Introduction

The term “hypophosphatasia” (HPP; OMIM #146300, #241500, #241510) comprises a group of rare inborn systemic metabolic diseases, characterized, and caused, by reduced, or absent, activity of the tissue-non-specific isoenzyme of alkaline phosphatase (TNSALP), encoded by the Alkaline Phosphatase, Liver (*ALPL*; OMIM * 171760) gene on chromosome 1p36.12.

Different clinical forms of the disease are specifically featured by different ages of onset, which are inversely associated with the clinical severity of the disease. Perinatal and infantile HPP are usually characterized by absent or extremely reduced TNSALP activity, due to homozygous or compound heterozygous loss-of-function mutations of the *ALPL* gene, and show very serious clinical phenotypes, mainly including severe rickets, thoracic dystrophy, respiratory failure due to chest deformities and lung hypoplasia, craniosynostosis, failure to thrive, and vitamin B6-responsive seizures, and are lethal in many cases [1,2]. Childhood HPP presents with an intermediate clinical phenotype generally characterized by rickets, bowing of the long bones, poorly healing fragility fractures, short stature, craniosynostosis, musculoskeletal pain, motor delays, muscle weakness, and a variety of symptoms related to dentistry, including adentulism, poor tooth alignment, tooth mobility, premature loss of deciduous teeth with intact tooth roots (due to impaired cementum formation), increased vulnerability to tooth decay and wear due to hypomineralized enamel and dentin, alveolar bone defects, and deeper periodontal pockets [1,2,3]. Adulthood-onset HPP typically presents with milder, heterogeneous signs and symptoms that usually manifest later in life. Initially, patients are commonly misdiagnosed with early-onset osteoporosis, due to the finding of low bone mass, recurrent fragility fractures, and bone pain [2,4]. However, a more in-depth anamnesis usually reveals a past clinical history of signs and symptoms typical of HPP, which often have already manifested during childhood and adolescence, such as rickets/osteomalacia, diffuse musculoskeletal pain, chronic weakness, recurrent metatarsal stress fractures, poorly healing fractures, chondrocalcinosis, premature tooth exfoliation, tooth mobility, early loss of deciduous and/or permanent teeth, recurrent caries, and periodontal disease [2,4].

HPP is a very complex and heterogeneous disease both from the point of view of clinical manifestations, which affect multiple organs and with variable clinical phenotypes even between affected members of the same family, and from the point of view of genetic inheritance, which can be autosomal dominant or autosomal recessive. Therefore, the diagnosing of HPP is still challenging in many cases, often missed or delayed by many years, especially for milder phenotypes or when a patient refers for the first time to a specialist who has no specific experience with rare diseases of bone and mineral metabolism. The diagnosis of HPP requires the integration of various factors: (1) an in-depth clinical and family history of the patient, (2) a radiological evaluation of the skeleton to identify specific bone and osteoarticular characteristics of the disease, (3) genetic testing of *ALPL*, which, if positive, can support the clinical findings, and (4) biochemical evaluation of specific parameters relating to HPP and of those useful for facilitating the differential diagnosis with other bone and mineral metabolism pathologies.

Recently, an HPP International Working Group, composed of a multidisciplinary team of experts in the field of this disease, conducted two systematic reviews of the literature and meta-analysis to assess major and minor criteria in the diagnosis of HPP in children and adolescents [1], and in adults [4]. Both the studies confirmed the repeated finding of total alkaline phosphatase (ALP) low enzymatic activity (adjusted for age and sex), measured in the blood, as the obligate diagnostic criterion for HPP at any age, and identified the elevation of natural substrates of TNSALP, in the blood and/or urine, as a major diagnostic criterium for HPP in children, adolescents, and adults.

**Figure 1:**
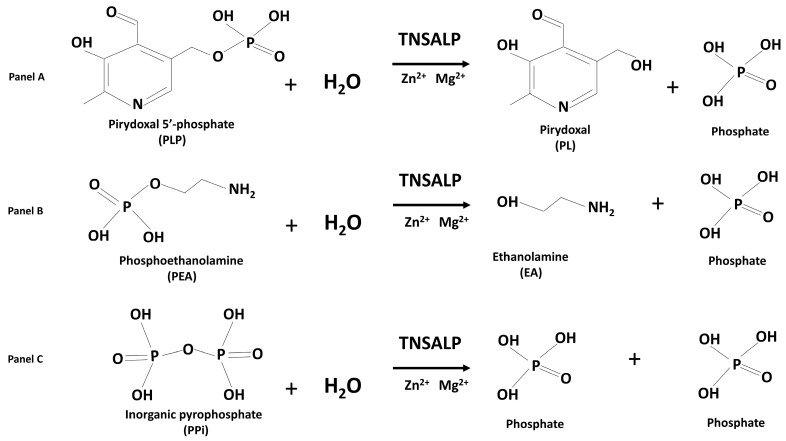
Dephosphorylation reactions catalyzed by TNSALP on its three main natural substrates, the pyridoxal 5′-phosphate (PLP) (Panel **A**), the phosphoethanolamine (PEA) (Panel **B**), and the inorganic pyrophosphate (PPi) (Panel **C**), which, in a basic pH environment in the presence of H_2_O, and with Zn^2+^ and Mg^2+^ as cofactors, are converted in pyridoxal (PL), ethanolamine (EA), and phosphate, respectively, by releasing a phosphate molecule.

TNSALP is expressed as a series of cell-specific isoforms, which differ in their post-translational modifications, and are active in bone, liver, kidney, and teeth. In cells, TNSALP is a homotetramer that is bound to the external surface of cell membrane as an ectoenzyme, anchored by a molecule of glycosylphosphatidylinositol. Circulating TNSALP is a homodimeric protein composed of two identical subunits, each one with an active site containing 3 metal-binding sites, two for ion zinc (Zn^2+^) and one for ion magnesium (Mg^2+^), two metal cations that are essential for the enzymatic catalytic activity. Enzymatic activity of circulating ALP is indicative of TNSALP activity in tissues. The bone-specific TNSALP isoform (BALP) present in the blood accounts for approximately 60% of serum ALP activity [5]. Germline loss-of-function mutations of the *ALPL* gene can variably affect the structure and function of TNSALP and are the cause of HPP in the great majority of patients, as heterozygous, composite heterozygous, or homozygous mutations. In an alkaline environment and in the presence of water molecules, the TNSALP catalyzes the reaction of the removal of phosphate groups (dephosphorylation) from various molecules. Its main substrates include the active form of vitamin B6 (pyridoxal 5′-phosphate; PLP), the phosphoethanolamine (PEA), and the inorganic pyrophosphate (PPi), which are converted in pyridoxal, ethanolamine, and phosphate, respectively, by releasing a phosphate molecule (Figure 1).

The visible consequence of inactivating mutations in TNSALP and/or of reduced enzymatic activity, in HPP patients, is the extracellular accumulation of its natural substrates, PLP, PEA, and PPi. PLP and PPi were found to be elevated in HPP patients and in animal models of HPP [6].

## 2. Biochemical Screening for ALP Activity in the Blood

Laboratory diagnosis of HPP is primarily based on repeated detections of low circulating ALP activity values. This finding is an obligatory diagnostic criterion for HPP both in children and adult patients [1,4], but not sufficient, alone, to biochemically define the disease.

The test is aimed at measuring the enzymatic activity of TNSALP, rather than its quantity in the blood.

Circulating ALP activity is assessed, on a serum sample, usually by an enzymatic kinetic photometric method, recommended by the International Federation for Clinical Chemistry (IFCC) [7], which measures the rate of transformation of a non-physiological substrate of TNSALP (para-nitrophenylphosphate; PNPP) into its dephosphorylated product (para-nitrophenol; PNP), by photometrically evaluating over time the absorbance of PNP at its specific wave length (around 400–405 nm under alkaline conditions) at 37 °C, which is proportional to ALP activity, with respect to a calibration curve. ALP activity is calculated as U/L, in which U is the International Unit; one unit of ALP is defined as the amount of enzyme that catalyzes the conversion of 1 µmol of PNPP, per minute, in the presence of H_2_O and Mg^2+^, to phosphate and PNP, under enzyme specific conditions.

Blood sample handling is of key importance for the result of the analysis, since errors in sample collection, transport, or processing can affect ALP activity levels. Hemolysis notably interferes with the assay, and it may increase ALP activity, falsifying the validity of the test. Indeed, ALP assay kit manufacturers recommend not processing hemolyzed samples.

The test is commonly performed on a serum sample. To obtain serum, whole blood samples have to be collected, in the morning after overnight fasting (no food or drink), preferably in the absence of any anticoagulant agent, mainly EDTA and oxalates, which, by chelating Mg^2+^ and Zn^2+^ ions, alters the activity of TNSALP and, therefore, interferes with the correct dosage of the same in the serum. Plasma testing can also be performed by collecting whole blood with selected anticoagulants (i.e., heparin, lithium, sodium, ammonium), not chelating cations. The collected whole blood sample must be centrifuged for about 10 min, preferably within one hour of collection using a refrigerated centrifuge at a temperature of +2–8 °C, to recover the serum or the plasma. The serum or plasma sample should be stored at −20 °C up to the analysis, strictly avoiding the freezing and thawing processes. The stability of ALP activity in human serum and plasma samples is about 7 days at 20–25 °C or 4–8 °C and up to 2 months when frozen at −20 °C.

However, even when blood sampling and analytical conditions of the ALP assay have been validated, it may be difficult to interpret the result of the test, particularly with respect to reduced values expected in HPP. Indeed, ALP activity is largely age- and gender-dependent, and the definition of correct reference values is difficult, especially in infants, children, and adolescents. Neonates have very high levels of ALP (usually 150–300 U/L) [5], children and teens have naturally elevated ALP activity due to the physiological process of bone growing and skeletal modeling and changes in the hepatobiliary system, and postmenopausal women may have increased ALP activity due to an accelerated bone turnover. Therefore, reference values have to be carefully adjusted, according to age ranges and sexes, and to the employed ALP assay, to distinguish between change due to physiological development and change due to the presence of disease. Commonly, age-dependent changes in ALP activity are interpreted using separate reference intervals for different age groups. However, this approach can lead to misinterpretation of the results, especially in individuals aged at the cut-offs of age groups, when upper and lower reference limits shift abruptly due to changes in age, and they may be classified differently, despite minimal biological differences, with the risk of having false positives or false negatives in diagnosis. To solve this issue, some authors suggested that age be considered as a continuous variable and use continuous reference ranges, rather than discrete age bins, via regression models or percentile curves, for children and adolescents [8,9]. In addition, in the field of HPP, for these individuals near the boundaries of age groups, it is suggested to incorporate additional factors (i.e., weight, height, and clinical signs) to better interpret the assay results.

A variety of biological or pathological conditions, pharmacological treatments, and endogenous or exogenous factors can influence circulating ALP activity values. Factors that can reduce or increase ALP activity in the blood are depicted in Table 1 and Table 2, respectively [10,11,12,13].

These factors should, therefore, be taken into account at the time of biochemical screening, when interpreting ALP levels. First of all, it is crucial to consider the patient’s overall clinical picture and perform an in-depth anamnesis, assessing the presence of specific clinical conditions or diseases, which are known to alter ALP activity, the therapeutic framework, and other relevant laboratory results. A general evaluation of metabolic functions, particularly hematological, hepatic, and renal functions, is strongly suggested, since their disfunctions can be responsible for alteration in ALP activity, due to incorrect synthesis or function of the liver and/or kidney isoforms of the enzyme. Eating a fatty meal before an ALP test may also cause a small increase in ALP activity values; thus, patients should be requested to avoid fatty meals in the 2–3 days before blood collection. In addition, since some drugs may lower or increase ALP levels, patients should be asked to suspend some days prior the analysis, if it is possible.

The bone-specific isoform of TNSALP (BALP), which is post-translationally modified and selectively expressed by osteoblasts, can be assayed by immuno-quantitative methods [enzyme-linked immunosorbent assay (ELISA) or Radioimmunoassay (RIA)] or by ELISA immuno-enzymatic assay (EIA). Concentration of circulating BALP does not directly allow the diagnosis of HPP, but it could help in the differential diagnosis with other rare metabolic bone diseases characterized by altered activity of osteoblasts.

## 3. Biochemical Screening of PLP Concentration in the Blood

Elevated values of PLP in the blood, in the absence of a vitamin B6 supplementation or a vitamin B6-rich diet, are another hallmark of HPP, included odonto-HPP. Elevated PLP in the presence of contemporary low ALP activity values is a distinctive biochemical profile found in many HPP patients, representing a diagnostic biochemical combination for HPP.

The term vitamin B6 comprises a group of three pyridine derivatives (pyridoxine, pyridoxamine, and pyridoxal) and their respective esters 5′-phosphate (pyridoxine 5′-phosphate, pyridoxamine 5′-phosphate, and PLP), which are taken in through food or supplementation and then are all converted to PLP in the liver. The PLP represents the biologically active form of vitamin B6 and it is the major form of circulating vitamin B6 primarily coupled to albumin. Only a minor fraction of PLP circulates freely in the blood.

In HPP patients, the reduced activity of TNSALP can result in increased levels of circulating PLP, in a way that seems to be directly proportional to the degree of reduction in enzymatic activity and to the severity of the disease. However, in most HPP patients, sufficient extracellular dephosphorylation of PLP to pyridoxal, by mechanisms other than TNSALP activity, seems to account for their normal vitamin B6 status [14].

For PLP measurement, a whole blood sample has to be preferred to plasma or serum, because it is less affected by short-term changes in the body’s acute phase response, which can alter serum PLP levels. A recent study, by Obeid R et al. [15], dosing PLP both on whole blood and serum samples from the same healthy volunteers, showed that concentrations of PLP in whole blood were 41% higher than those in serum.

Pre-analytic conditions, for both sample collection and processing, can significantly influence the result of the analysis, and specific precautions must be taken into consideration. Whole blood has to be collected in the presence of anticoagulant (sodium or lithium heparin, EDTA, or K_2_ EDTA), in the morning after overnight fasting, since PLP levels are affected by the nutritional status, and a high carbohydrate intake can decrease circulating PLP values. PLP is decomposed by light or by oxidizing substances, and it decreases in samples stored at room temperature due to dephosphorylation to pyridoxal; therefore, the collected blood sample has to be managed while strictly protected by light (by being stored in amber or dark test tubes) immediately after sampling and during the entire analysis performance, and be stored at −20 °C, up to the analysis, avoiding freezing and thawing processes.

Concentration of circulating PLP is usually determined by high-performance liquid chromatography (HPLC) with a fluorescence detector, after deproteinization of the blood sample, and a specific microchemical reaction (derivatization) to alter the structure of the analyte to facilitate its isolation and separation, and, most of all, to permit the fluorometric detection. Some laboratories detect PLP by using liquid chromatography–tandem mass spectrometry (LC-MS/MS) for precise measurement, often in plasma.

PLP can be measured as ng/mL or nmol/L, and reference ranges are commonly defined according to the employed assay.

Dietary habits, the presence of some clinical conditions, smoking, chronic abuse of alcohol, and the administration of some medications that can bind to and inactivate PLP or interfere with vitamin B6 metabolism (Table 3) [16,17], at the time of blood sampling, or variations in serum albumin levels can alter levels of circulating PLP [16], and, thus, they should be taken into account in the interpretation of test results.

In addition, PLP levels correlate with aging, showing decreasing levels in older adults. Conversely, high blood PLP levels may occur in postmenopausal females where accelerated bone resorption increases serum inorganic phosphate (Pi) levels, which in turn inhibits 50% of TNSALP enzymatic activity [17].

## 4. Biochemical Screening of PEA Concentration

PEA is both a precursor of phospholipid biosynthesis and a product of their catabolism. Elevated levels of PEA in urine have been demonstrated in some adults and children with HPP, and, thus, they can support a diagnosis of HPP. However, this elevation is not specific to HPP and can occur in other metabolic bone disorders. Moreover, not all HPP patients have urinary PEA values above the reference ranges, especially those who have later onset and milder disease [18,19,20].

PEA excretion levels in urine are conditioned by dietary habits and patient age, and it follows a circadian rhythm [14]; to reduce the influence of the last factor, and obtain a reliable measurement, a 24 h urine sample is ideal. High-protein diets, especially from meatless sources, can increase urine excretion of PEA, causing “false positives” in testing. The same can occur with a deficiency of magnesium and/or manganese, two enzymatic cofactors in the metabolism of PEA, which can contribute to elevated levels of urinary PEA. Conversely, malnutrition, deficiency of vitamin B12 or folic acid, or poor dietary intake of proteins can lead to reduced levels of PEA. Celiac disease has been linked to increased PEA levels in the urine. Liver and kidney diseases and hypertension can also influence PEA excretion. Moreover, since gastrointestinal microbiome imbalance can influence levels of ethanolamine, the biological precursor of PEA, this condition can be responsible for variations in PEA values.

Measurement of PEA in urine is not routinely assayed in the clinical management of HPP; this analysis is usually performed only in few “inborn error” laboratories, through chromatography methods, such as HPLC combined with different detectors (UV–visible, fluorimeter, or mass spectrometer).

A derivatization reaction is needed to enable the separation of the target compound from the hydrophilic pool of phosphorous metabolites in urine. A simple one-step derivatization procedure with fluorenylmethyloxycarbonyl chloride (fmoc-Cl), performed under basic pH conditions, makes the non-aromatic amine PEA detectable by the detector in the UV–visible spectrum.

HPLC, associated with an element-selective inductively coupled plasma tandem mass spectrometer (ICP-MS/MS) as detector, allows highly specific detection and quantification of the phosphorus component of PEA, and it has been recently tested for application also in human urine [21]. However, this technique is complex, time-consuming, and expensive to be easily applied in clinical analyses.

Pre-analytic conditions for biological sample collection and storage are important to grant the validity of the test result. Urinary PEA can be measured both on a spot urine sample collected in the morning after overnight fasting or a 24 h urine sample. A 24 h urine sample allows to mitigate the fact that urinary PEA excretion levels can be influenced by circadian rhythm. However, since 24 h urine collection is a more complex procedure for the patient, spot urine sampling is often preferred. Collected urine samples have to be stored at −20 °C or −80 °C before the analysis, avoiding freezing and thawing processes.

Urinary PEA (either on the urine spot or urine 24 h samples) is normalized to the urinary creatinine content to account for variations in urine concentration. Measurement units, as well as reference values, vary based on the analytical laboratory, being reported as μmol PEA/g creatinine, nmol PEA/mg creatinine, mg PEA/g creatinine, or mmol PEA/mol creatinine. In 1988, MacFarlane et al. [22] reported an upper limit of normal of 11.3 μmol PEA per millimole of creatinine, by screening a 24-year-old woman with HPP and 22 members of her family, by using an automated chromatographic method for amino acids. Two more recent studies, both testing urinary PEA by ion exchange chromatography, reported reference ranges for PEA in urine to be 0.0–15.0 mmol/mol creatinine [23] or 0–27 nmol/ mg creatinine [20], both without specifying the population data on which they are based.

## 5. Biochemical Screening of PPi Concentration

The overall extracellular values of PPi derive from a variety of biochemical reactions in human metabolism that generate PPi (ATP hydrolysis, DNA and RNA polymerization, cyclic AMP formation, and enzymatic activation of fatty acids to form their coenzyme A esters) and by enzymatic reactions that consume it as a substrate (cyclases, hydrolases, and ligases). Normally, the extracellular PPi/Pi ratio is maintained constant to prevent the ectopic calcification of soft tissues. In HPP patients, the reduced TNSALP activity reduces the rate of PPi degradation to Pi, consequently leading to increased circulating levels of PPi. PPi levels in urine were also found elevated in a percentage of patients with HPP, even if it can be normal in mildly affected individuals.

Laboratory assays for measuring PPi in urine or plasma are not currently employed in the clinical practice, being exclusively a research technique; an internationally standardized PPi assay is still missing. Many of the studies describing methods for the measurement of PPi in human fluids are older than 30–40 years. However, they represent the basis of analytical methodologies currently employed for PPi dosage.

The evaluation of extracellular PPi in human fluids is extremely difficult and it is complicated by a variety of altering factors. PPi is subjected to variable and often extensive hydrolysis during sampling and analysis. Minimizing the overall time intercurrent between blood taking and plasma retrieval, performing sample handling on ice and using a refrigerated centrifuge, freezing plasma and urine at −80 °C immediately after their collection, and performing all the analytical procedure while maintaining the sample in ice, reduce the risk of PPi degradation and of the underestimation of actual values. Blood has to be strictly collected in the presence of anticoagulants lithium heparin, since, without a heparin-mediated blockage of the coagulation cascade, activated platelets release about half of their intracellular PPi amount during coagulation. Indeed, it has been reported that serum samples contain PPi levels 2–3 times higher than plasma [24].

In the plasma, concentrations of PPi are lower than in urine, and other compounds interfere with its chemical determination. Moreover, since platelets are rich in PPi, the release of PPi from platelet lysis at the time of blood sampling and plasma processing can result in falsely elevated plasma levels. Intracellular PPi concentration in platelets is about 800-fold higher than in plasma; thus, even a minimal amount of platelet lysis can lead to overestimating the actual levels of circulating PPi. Platelets have to be removed immediately after plasma retrieval, either by ultracentrifugation or using a 300,000 Dalton molecular weight cut-off filter under 2000× *g* centrifugation for 20 min at 4 °C [25,26], preferably filtering a small volume of plasma (up to 1 mL) per filter to avoid the possibility of coagulation.

The test to measure extracellular PPi was first described in 1964 on urine samples [27], and then modified in 1971 and applied to human plasma [28], consisting in an isotope dilution method, using ^32^P-labeled pyrophosphate that was added to whole blood at the time of sample collection, followed by the recovery of plasma by centrifugation, plasma deproteinization by ultrafiltration, two coprecipitations of PPi with calcium phosphate, treatment with a cation exchange resin to remove calcium and nucleotides, and finally the separation of PPi from other phosphate compounds by ion exchange chromatography through an anion exchange resin. Circulating PPi initial concentration was calculated with respect to the radioactivity of PPi eluted from the column.

Since then, various techniques were used to measure PPi in plasma and/or urine.

One commonly employed enzymatic method is a three-step assay that uses uridine-5′-diphosphoglucose (UDPG) as a substrate of UDPG pyrophosphorylase, suitable for a small volume of deproteinized ultrafiltrates human fluids [29]. During the first step, this enzyme catalyzes the reaction of UDPG and PPi to produce glucose-1-phosphate and uridine triphosphate. Then, a second enzyme, phosphoglucomutase, converts the glucose-1-phosphate into glucose-6-phosphate. Finally, a third enzyme, the glucose-6-phosphate dehydrogenase, converts glucose-6-phosphate into 6-phosphogluconate in the presence of NADP+, which is reduced to NADPH. The final amount of NADPH produced is measured spectrophotometrically at 340 nm to determine the initial concentration of PPi. This method requires the initial separation of PPi from plasma by ultrafiltration to avoid the influence of inhibitory ions, and the deproteinization of plasma to remove endogenous enzymes that use PPi as a substrate [30]. The need for plasma ultrafiltration makes this approach limited to very few clinical laboratories that have access to an ultracentrifuge.

Other assays use enzymatic reactions (i.e., ATP sulfurylase) to convert PPi to ATP that is then detected by a luciferase/luciferin luminescence reaction, in a semiquantitative manner, by using a PPi standard curve as a reference substrate to calculate the initial amount of PPi in the biological sample [26,31,32]. This is a complex and time-consuming technique that requires one to measure initial extracellular ATP present in the blood and then subtract its concentration from the final ATP dosed.

Recently, commercial standardized assays to measure PPi in human fluids have been developed. They are based on the application of composite enzymatic reactions by utilizing an enzyme mix and probe that generate a stable product, directly proportional to the initial PPi, that can be quantified by colorimetric or fluorometric detectors, or on the use of a specific fluorogenic PPi-sensor in which the presence of PPi in the reaction mixture results in the production of a fluorescent product proportional to the PPi present.

The universal definition of plasma or urine PPi standard ranges does not exist due to the unavailability of a validated dosing method. The PPi normal range in plasma is narrower than that in urine. Different studies, performed by using different dosage methods, have placed the reference values of PPi in plasma in rather similar ranges, overall ranging from 0.16 to 5.9 μM [26,28,29,30,33,34,35,36]. Plasma PPi levels showed no significant difference between males and females, both in adults and children [26,28], or different times of blood sampling over the day [28], even if they were found to be elevated at night [37]. Children less than 15 years old showed faintly higher plasma PPi levels than adults over 20 years [28]. This finding was not confirmed in a more recent study, which found plasma PPi values in patients <18 years comparable to those in adults, also without significant differences between different pediatric age groups [26]. Plasma PPi is lower during fasting and correlate with Pi levels in non-fasting individuals [37].

Urine PPi values are dependent on time of day and fasting state at the time of collection, being lower in fasting subjects and at night [27,37]. PPi urine excretion strongly correlates with Pi and it is directly dependent on the oral intake of Pi [37,38]. Moreover, starting from the fifth decade of life, urine PPi concentration was shown to decrease rapidly [39]. The definition of the standard range for urine PPi is even more complex than for plasma, since variable values have been reported in different studies. In 1988, MacFarlane et al. [22] reported an upper limit of normal of 5 μmol PPi per millimole of creatinine, by screening a 24-year-old woman with HPP and 22 members of her family, by using the UDPG pyrophosphorylase-based method. More recently, a reference range for PPi in spot urine was reported as 7.7–56.7 mmol/mol creatinine in children [23,40].

## 6. Additional Screenings for the Differential Diagnosis

The biochemical evaluation of other specific parameters in the blood, not directly associated with HPP, can be of support in the differential diagnosis of HPP with respect to other rare metabolic bone diseases.

Serum calcium and phosphate, which are usually reduced in nutritional rickets and other forms of rickets, and phosphate, which is usually reduced in X-linked hypophosphatemic rickets, are both usually elevated, or normal in milder cases, in HPP patients. Parathyroid hormone (PTH), which is usually elevated in rickets (included X-linked hypophosphatemic rickets) and normal in osteogenesis imperfecta, is commonly reduced, or, more rarely normal, in HPP patients.

## 7. Methods

This review synthesizes and discusses biochemical analyses that can be performed to help in the diagnosis of HPP. The literature studies on Pubmed have been researched by the authors to identify relevant studies in this field, by using combinations of the following free-text keywords: hypophosphatasia, biochemical analyses/screenings, diagnosis, alkaline phosphatase, alkaline phosphatase activity, ALP, TNSALP, active vitamin B6, pyridoxal 5′-phosphate, PLP, phosphoethanolamine, PEA, inorganic pyrophosphate, and PPi.

## 8. Conclusions

The possibility to simultaneously assess ALP, PLP, PEA, and PPi, in a patient with suspected HPP, is important to facilitate and expedite the diagnosis of HPP, and support clinicians in the clinical and therapeutic management of patients.

Furthermore, screening for these four parameters can be useful not only in the diagnostic setting, but also to ensure response monitoring to enzyme replacement therapy, which is now available for HPP.

A study evaluating the effects of enzyme replacement therapy in 21 HPP adult patients with pediatric onset of the disease showed a significantly increased ALP activity and reduced urine PEA/creatinine ratios with respect to the baseline, through the 24 months of treatment [41].

Changes in plasma PLP and of plasma and/or urine PPi have been used as response outcomes in clinical trials and observational studies evaluating the efficacy of enzyme replacement therapy in HPP patients. In a Phase 2a, open-label studies on enzyme replacement therapy in adults with pediatric-onset HPP, changes occurring in plasma concentrations of PPi and PLP from the baseline were evaluated as primary and secondary outcome responses to treatment, respectively [42]. The pharmacological treatment resulted in a reduction in both plasma PPi and PLP. Different dose regimens showed significant differences between least squares mean changes from the baseline, both in plasma PPi and in plasma PLP. A treatment group showed the over-suppression of PPi, suggesting that the administered dose may be too high, while another group of treatment had PLP concentrations above the upper limit of normal, suggesting that the administered dose may be too low to effectively reduce PLP within the normal values. Another study confirmed the decrease in plasma PPi to the reference range after the first week of treatment with enzyme replacement therapy, and also showed urine PPi (corrected by urine creatinine) to decrease in parallel and with a high correlation to plasma PPi [43].

A recent Phase 1 study on a second-generation enzyme replacement therapy for TNSALP measured plasma concentrations of PPi and PLP, and the PLP/PL ratio overtime, as drug efficacy outcomes [44]. This trial showed reductions in plasma PPi and PLP concentrations of ≥60% and ≥69%, respectively, following drug administration, with the magnitude and duration of these reductions appearing to be dose-dependent. Both PPi and PLP values were maintained within the normal ranges during the treatment period, but gradually returned to baseline-elevated values after the interruption of treatment.

## Figures and Tables

**Table 1:** Endogenous and exogenous factors that can determine reduced values of ALP activity measured in the blood (hypophosphatasemia).

**Nutritional conditions**
Zinc and/or magnesium deficiency
Vitamin D excess
Vitamin C deficiency (scurvy)
Marked nutritional impairments (anorexia, prolonged fasting, malnutrition)
Protein/calorie deficiency
Alcohol consumption
**Diseases and clinical conditions**
Hypophosphatasia
Hypothyroidism
Hypoparathyroidism
Cushing’s syndrome
Hereditary hypophosphatemia
Wilson’s disease
Corticoid-induced osteoporosis
Chronic renal osteodystrophy
Multiple myeloma
Severe osteogenesis imperfecta (mostly in newborns and infants)
Cleidocranial dysplasia (severe cleidocranial dysplasia is the most likely disorder to be confused with HPP)
Celiac disease
Achondrodysplasia
Severe forms of anemia (pernicious anemia, aplastic anemia, etc.)
Inflammatory bowel diseases (ulcerative colitis, Crohn’s disease) and intestinal infections
Milk–alkali syndrome (MAS)
**Pharmacological treatments**
Prolonged bone resorption treatments (bisphosphonates and denosumab)
Estrogens, hormonal contraceptives, anabolic steroids, and methyltestosterone
Estrogen replacement therapy in postmenopausal women
Certain diuretics (acetazolamide, furosemide, ethacrynic acid, and chlorothiazide)
Bezafibrate, clofibrate, and other fibric acid derivatives used to treat hyperlipidemia (particularly on the liver isoform of TNSALP)
Propanolol (particularly in patients with secondary hyperparathyroidism due to chronic kidney disease, or in cases of postmenopausal osteoporosis)
**Other factors**
Intake of radioactive heavy metal
Recent massive transfusion of whole blood or plasma
Cardiac surgery or cardiopulmonary bypass
Posthepatic resection or liver transplantation

**Table 2:** Endogenous and exogenous factors that can determine increased values of ALP activity measured in the blood (hyperphosphatasemia).

**Nutritional conditions**
A dietary habit stressing the liver (fatty or fried foods, excessive alcohol, processed foods, sugary drinks, and foods high in refined carbohydrates)
Vitamin D deficiency
**Diseases and clinical conditions**
Hyperthyroidism
Hyperparathyroidism
Liver disease or dysfunction (cirrhosis, drug-induced liver failure, liver cancer, infectious and non-infectious hepatitis)
Cholangiocarcinoma (bile duct cancer)
Gallstones, cholelithiasis, biliary tract obstruction
Cholecystitis (inflammation of the gallbladder)
Kidney tumors (including renal cell carcinoma)
Prostate cancer
Lymphomas or other neoplasms of the lymphatic system
Myelofibrosis
Bone tumors (osteosarcoma, chondrosarcoma)
Bone metastases and/or liver metastases
Paget’s disease of bone
Rickets or osteomalacia
Mononucleosis, current bacterial infection, sepsis
Sarcoidosis
**Pharmacological treatments**
Bone anabolic drugs (teriparatide)
Antibiotics (penicillin derivatives, nitrofurantoin, erythromycin, aminoglycosides, sulfonamides, and trimethoprim–sulfamethoxazole. Rifampicin selectively increases bone alkaline phosphatase)
Glucocorticoid (corticosteroid) therapy (prednisolone)
Antiepileptic drugs (carbamazepine, phenobarbital, phenytoin, and valproic acid)
Antihistamines (cetirizine)
Disease-modifying agents (penicillamine and gold salts)
Cardiovascular drugs (captopril, diltiazem, felodipine, verapamil, quinidine, and flutamide)
Anti-hypertension drugs (verapamil, captopril, methyldopa)
Tricyclic antidepressants and monoamine oxidase inhibitors
Anti-psychotic drugs (phenothiazines, chlorpromazine)
Diabetes drugs (tolbutamide, chlorpropamide, and tolazamide)
Some chemotherapy drugs (carboplatin and docetaxel)
Allopurinol
Methimazole
Disulfiram
Phenylbutazone
**Other factors**
Puberty
Pregnancy, particularly in the third trimester
Fracture healing or recent orthopedic surgery

**Table 3:** Endogenous and exogenous factors that can modify PLP values measured in the blood.

**1. Factors that can increase blood PLP**
**Nutritional conditions**
Vitamin B6-rich diet
Supplementation with vitamin B6
**Diseases and clinical conditions**
Hypophosphatasia
**Other factors**
Menopause
**2. Factors that can decrease blood PLP**
**Nutritional conditions**
Malabsorption or malnutrition
Chronic abuse of alcohol
**Diseases and clinical conditions**
Chronic inflammatory status (acute phase of rheumatoid arthritis)
Nerve compression disorders (carpal tunnel and tarsal tunnel syndromes)
Chronic kidney diseases and liver diseases (due to increase in TNSALP activity)
Cardiovascular diseases (atherosclerosis, early myocardial infarction, early stroke, recurrent thromboembolism, deep venous thrombosis)
Diabetes
Genetic disorders of the pyridoxamine 5′-phosphate oxidase (*PNPO*) and pyridoxal phosphate-binding protein (*PLPBP*) genes
Inflammatory bowel diseases
**Pharmacological treatments**
D-penicillamine
Hydralazine
Isoniazid
Cycloserine
Pyrazinamide
Phenelzine
Thiamphenicol
L-dopa
Theophylline
Antiepileptic drugs (Progabide)
Oral contraceptives
**Other factors**
Aging
Pregnancy
Smoking (proportionally to the numbers of cigarettes smoked per day)
Renal transplant

## Data Availability

No new data were created or analyzed in this study. Data sharing is not applicable to this article.

## Abbreviations

The following abbreviations are used in this manuscript:

TNSALP	Tissue-non-specific alkaline phosphatase
HPP	Hypophosphatasia
ALPL	Alkaline phosphatase, liver (gene)
ALP	Alkaline phosphatase
PLP	Pyridoxal 5′-phosphate
PEA	Phosphoethanolamine
PPi	Inorganic pyrophosphate
IFCC	International Federation for Clinical Chemistry
PNPP	Para-nitrophenylphosphate
PNP	Para-nitrophenol
BALP	Bone alkaline phosphatase
ELISA	Enzyme-linked immunosorbent assay
RIA	Radioimmunoassay
EIA	ELISA immuno-enzymatic assay
HPLC	High-performance liquid chromatography
LC-MS/MS	Liquid chromatography–tandem mass spectrometry
UDPG	Uridine-5′-diphosphoglucose
